# Prevalence and factors associated with low bone mineral density in Saudi women: a community based survey

**DOI:** 10.1186/1471-2474-15-5

**Published:** 2014-01-08

**Authors:** AlJohara M AlQuaiz, Ambreen Kazi, Salwa Tayel, Shaffi Ahamed Shaikh, Abdullah Al-Sharif, Saleh Othman, Fawzia Habib, Mona Fouda, Riad Sulaimani

**Affiliations:** 1 Princess Nora Bent Abdallah Women Health Research Chair, College of Medicine, King Saud University, PO Box 231831, Riyadh 11321, Kingdom of Saudi Arabia; 2 Department of Family & Community Medicine, College of Medicine, King Saud University, Riyadh, Kingdom of Saudi Arabia; 3 Council of Co-operative Health Insurance, Ministry of Health, Riyadh, Kingdom of Saudi Arabia; 4 Department of Nuclear Medicine, College of Medicine, King Saud University, Riyadh, Kingdom of Saudi Arabia; 5 Departement of Obstetrics & Gynecology, College of Medicine, Taibah University, Al Medina, Al Munawara, Kingdom of Saudi Arabia; 6 Department of Medicine-Endocrinology, College of Medicine, King Saud University, Riyadh 12371, Saudi Arabia

**Keywords:** Low bone mineral density, Women, Dietary factors

## Abstract

**Background:**

Low bone mineral density (BMD) is a public health issue in Saudi Arabia. This study measured the prevalence and factors associated with low BMD in Saudi women in Riyadh, Saudi Arabia.

**Methods:**

A cross sectional study using two stage cluster sampling technique was conducted in Riyadh, 2009. Thirty clusters, each comprising of 300 houses were randomly chosen and from each cluster 38–40 households were selected to identify 1150 women of >40 years. Women were invited to primary health care center for filling of self-administered questionnaire (n = 1069) comprising of sociodemographic, health, diet and physical activity variables. 1008 women underwent screening for low BMD using the quantitative ultrasound technique. 535 (53%) women with positive screening test were referred to King Khalid Hospital for Dual X-ray Energy absorptiometry (DXA).

**Results:**

362 women underwent DXA and 212 (39.6%) were screened low BMD either at lumbar spine or femur neck. Mean age of women was 55.26(±8.84) years. Multivariate logistic analysis found; being aged 61 to 70 years (OR 2.75, 95% CI: 1.32-1.48), no literacy (OR 2.97, 95% CI:1.44 - 6.12) or primary education (OR 4.12, 95% CI:2.05-8.29), history of fractures (OR 2.20, 95% CI:1.03- 4.69) and not drinking laban(diluted yogurt) (OR 2.81, 95% CI:1.47- 5.37) significantly associated with low BMD.

**Conclusions:**

Women with low level of education, who do not drink laban and had history of fractures were at high risk of low BMD.

## Background

Low bone mineral density (BMD) manifesting as fragile bones mainly comprise of osteoporosis and osteopenia [1]. Prevalence of low BMD varies according to age, sex, ethnicity and type of skeletal bone [1]. According to global estimates, about 200 million women suffer from osteoporosis worldwide [2]. The American 2005–2008 National Health and Nutrition Examination Survey found 50% of women over 50 years of age suffering from low BMD [3]. Low BMD is a public health issue in Saudi Arabia and prevalence of lumbar and femur osteopenia ranges from 7% to 43.4% and osteoporosis from 2.5 to 46.7% [4-7].

National Osteoporotic foundation, USA and the National consensus group on osteoporosis for the Middle East and North Africa has indicated; menopause, low physical activity, family history of fractures, personal history of fracture as an adult, cigarette smoking, alcohol drinking, thin build, use of oral gluco-corticosteroid therapy for 3 months or more or having history of rheumatoid arthritis, thyroid disease, liver disease as major risk factors for low BMD [7-9]. In addition to above, Rouzi et al. in a prospective cohort study on healthy Saudi postmenopausal women found a combination of factors (*age, physical activity, hand grip, bone mineral density, dietary calcium intake, serum 25 (OH)D levels and history of falls)* to contribute significantly to osteoporosis-related fractures [10].

Low BMD leads to limitations in mobility along with high medical cost. To date, the gold standard technique for diagnosing low BMD is the dual energy x-ray absorptiometry (DXA) which has high predictive validity (sensitivity and specificity) [11]. Number of hospital based and few community based studies have measured risk factors for fractures and low BMD in Saudi Arabia; however, these studies have reported limitations in generalizability and quality [3-6]. In order to improve primary prevention against low BMD it is important to identify the few, most important risk factors through a community based study. The objective of this study was to measure the prevalence of low BMD using the DXA technique and identify the associated factors in Saudi women in Riyadh.

## Methods

**Figure 1:**
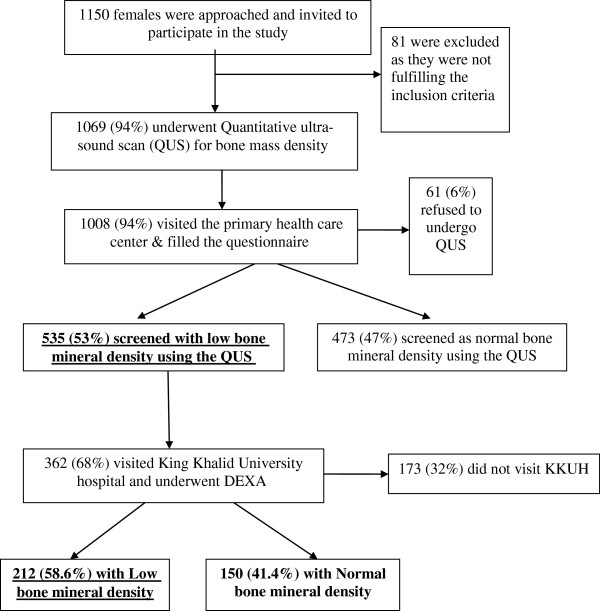
**Enrollment flow chart of Saudi women (>40 years) in Riyadh, Saudi Arabia**.

This was a community based household cross-sectional study conducted in Riyadh during April-May, 2009. Two stage cluster sampling technique was followed. Riyadh is divided into five administrative regions and one major primary health care center (PHCC) was selected from each region. The catchment population of PHCC served as a cluster. It is assumed that on an average there is one woman over forty years per Saudi family, therefore 230–240 households were randomly selected from each cluster to reach a sample size of 1150. Eligible women were invited to PHCC for filling of self-administered questionnaire and screening for low BMD using the Quantitative ultrasound (QUS) technique. Flow chart representing the enrollment and number of participants is given as Figure 1.

Questionnaire was designed based on previous identified factors and other biological plausible factors leading to low BMD. Information was collected on variables related to socio-demographic characteristics (age, education, occupation, marital status), sun exposure (time spent under the sun), present and past medical illnesses, obstetric & gynecological history (menstrual status, number of pregnancies and children, contraceptive use), personal history of fracture as an adult on trivial (minor) fall, family history of fractures, cigarette smoking, intake of dietary items; low fat milk, low fat cheese, low fat laban (diluted yogurt), meat, fish, vegetables, fruits, tea, coffee and physical activity (walking, climbing, swimming). All questions were phrased clearly for the participants to understand. Diet and physical activity was asked pertaining to last one week. Height and weight was measured through standard procedure. Those who had difficulty in reading or writing were interviewed by a trained research assistant.

### Inclusion & exclusion criteria

Saudi females, >40 years of age and living in Riyadh city were included in the study. Women who self reported secondary causes of low BMD, such as hyperthyroidism, hyperparathyroidism or liver disease were excluded from the study.

### Measurement of bone mineral density through quantitative ultrasound

QUS was done by scanning the calcaneum of the left foot using the Achilles machine (Lunar, General Electric, Madison, Wis.). The Achilles device is a water-based system, using fluid coupled through transmission in a temperature-controlled water bath (37 °C). The cutoff point for positive screened case was t score < −1 at the calcaneum heel bone [12]. Two QUS machines (from same manufacturer) were used and both machines were calibrated every morning according to the manufacturer’s instructions. Out of 1008 women who underwent QUS, 535 (53%) were screened positive. They were further referred to King Khalid University & Hospital (KKUH) for Dual energy x-ray absorptiometry (DXA) measurements.

A workshop was conducted to train the research assistants on the use of Achilles machine. Study protocol was approved by the Institutional Review Board (IRB) of the King Saud University.

### Dual energy x-ray absorptiometry

Out of 535 referred women, 362 (68%) underwent DXA. BMD was measured at the anterio-posterior spine L1-4 and Dual Femur Total (using GE prodigy, Lunar GE, Wisconsin USA). The quality control procedure for the machine was carried out every morning according to manufacturer’s protocol. All patients had the test performed, processed and finalized for reporting on the same day. The automatic region of interest (ROI) was used in all procedures to calculate the BMD at lumbar spine and femoral neck. The manual adjustments in ROI were made for the lumbar spine when necessary, for example, in cases of severe scoliosis. In addition, women were asked to give their blood samples in the laboratory (KKUH) for measurement of serum calcium and 25(OH) vitamin D levels.

### Statistical analysis

The stiffness index measured using the QUS was converted to corresponding t-scores with cut off of ≤ −1 as positive for LBMD. Similarly, low BMD measured using DXA was defined according to WHO cut off of t score ≤ −1[13] (includes both osteopenia and osteoporosis) present either at lumbar spine or femur neck to develop a dichotomous variable. Mean and standard deviation was calculated for continuous and proportions for categorical variables. All variables significant on univariate analyses and having biological plausibility were entered in multivariate logistic regression model using forward step modeling. Adjusted odds ratios and 95% confidence intervals were calculated. Significance was taken as α ≤ 0.05.

## Results

The socio demographic characteristic of women undergoing screening using the QUS are presented in Table 1. Mean age of women was 55.64 (±8.84) with range from 41 to 85 years. Mean serum calcium and 25 (OH) vitamin D levels in low BMD and normal group were 2.22 (±0.16) *vs* 2.28 (±0.17)mmol/L and 42.20 (±28.43) *vs* 38.45 (±23.54)nmol/L respectively. Further division of vitamin D into ≥75, 74–50 and <50 nmo/L category found 30% *vs* 18%, 37% *vs* 20% and 136% *vs* 116% (p < 0.22) women in low BMD and normal BMD groups respectively.

**Table 1:** Characteristic of Saudi women screened for low bone mineral density using quantitative ultrasound technique in Riyadh, Saudi Arabia

**Characteristic**	**Women screened as positive for low BMD using QUS (n%)**	**Women screened as negative for low BMD using QUS (n%)**	**P value**
**Age (in years)n = 1005**			
41 to 50	166 (31.1)	212 (45)	<0.01
51 to 60	228 (42.7)	185 (39.3)	
61 to 70	103 (19.3)	55 (11.7)	
70 and above			
**Marital Status n = 995**			
Single*	50 (9.5)	41 (8.8)	<0.01
Married	308 (58.2)	327 (70.2)	
Widow	171 (32.3)	98 (21)	
**Education n = 1008**			
University and above	25 (4.7)	31 (6.6)	<0.01
Intermediate& secondary	30 (5.6)	48 (10.1)	
Primary	24 (4.5)	41 (8.7)	
Illiterate	456 (85.2)	353 (74.6)	
**Occupation n = 962**			
Doctor	1 (0.2)	5 (1.1)	0.08
Teacher/Administrator	45 (8.8)	51 (11.4)	
Housewife	467 (91)	393 (87.5)	
**Husband Occupation n = 748**			
Doctors/teachers	32 (8.5)	26 (7)	0.36
Business man	98 (26)	104 (28)	
Military	107 (28.4)	88 (23.7)	
Administration	140 (37.1)	153 (41.2)	
**Type of House n = 965**			
Villa	390 (76.2)	342 (75.5)	0.95
Apartment	74 (14.5)	66 (14.6)	
Small house	48 (9.4)	45 (9.9)	
**Body Mass Index n = 981**			
Normal	60 (11.5)	30 (6.6)	<0.01
Overweight	149 (28.5)	113 (24.7)	
Obese	314 (60)	315 (68.8)	
**Exposure to sunlight per week n = 966**			
All the time	130 (25.4)	104 (22.9)	0.29
Twice-thrice per week	119 (23.2)	97 (21.4)	
Occasionally	208 (40.6)	212 (46.7)	
Not at all	55 (10.7)	41 (9)	
**Duration of exposure n = 785**			
Sixty minutes	7 (1.6)	4 (1.1)	0.13
Half an hour	103 (24.1)	66 (18.5)	
Fifteen minutes	318 (74.3)	287 (80.4)	
***Obstetric history***			
**Age at menarche n = 978**			
≤ than 11 years	141 (27.3)	119 (25.8)	0.82
12 to 14 years	303 (58.7)	274 (59.3)	
≥15 years	72 (14)	69 (14.9)	
**Menstrual status n = 920**			
Regular	170 (36)	178 (39.7)	0.23
Peri-menopausal**	168 (35.6)	136 (30.4)	
Menopause	134 (28.4)	134 (29.9)	
**Number of pregnancies n = 963**			
No pregnancy	10 (2)	20 (4.4)	0.08
Pregnant 1–4 times	66 (12.9)	62 (13.7)	
Pregnant >5 times	436 (85.2)	369 (81.8)	
**History of breast feeding n = 956**			
Not at all***	16 (3.1)	20 (4.5)	<0.01
For <6 months	119 (23.4)	140 (31.3)	
For >6 months	374 (73.5)	287 (64.2)	
**Use of Contraceptive pills**			
**n = 947**			
Yes	272 (54)	208(47)	0.03
No	232 (46)	235(53)	
**Duration of contraceptive pill use n = 504**			
Less than 5 years	144 (57.8)	175 (68.6)	<0.01
5 to 10 years	78 (31.3)	47 (18.4)	
More than 10 years	27 (10.8)	33 (12.9)	
***Past medical history***			
**Diabetic on insulin n = 1008**			
No	337 (63)	283 (59.8)	0.30
Yes	198 (37)	190 (40.2)	
**Past history of fractures on trivial fall n = 954**			
No	442 (86.8)	398 (89.4)	0.13
Yes	67 (13.2)	47 (10.6)	
***Family History***			
**History of Osteoporosis in the family n = 870**			
No	457 (98.3)	389 (96)	0.04

Yes	8 (1.7)	16 (4)	
**Family history of fracture on trivial fall n = 846**			
No	449 (98.5)	379 (97.2)	0.19
Yes	7 (1.5)	11 (2.8)	
***Physical Activity***			
**Walking n = 1008**			
Yes	274 (51.2)	258 (54.5)	0.29
No	261 (48.8)	215 (45.5)	
**Climbing Stairs n = 1008**			
Yes	28 (5.2)	25 (5.3)	0.97
No	507 (94.8)	448 (94.7)	
**Swimming n = 1008**			
Yes	11 (2.1)	6 (1.3)	0.33
No	524 (97.9)	467 (98.7)	
***Dietary Habits***			
**Milk (200 ml) n = 1008**			
Yes	420 (78.5)	397 (83.9)	0.03
No	115 (21.5)	76 (16.1)	
**Laban (200 ml) n = 1008**			
Yes	451 (84.3)	410 (86.7)	0.28
No	84 (15.7)	63 (13.3)	
**Cheese (slice = 100gms) n = 1008**			
Yes	421 (78.7)	399 (84.4)	0.02
No	114 (21.3)	74 (15.6)	
**Fish slice(any type) n = 1008**			
Yes	330 (61.7)	323 (68.3)	0.03
No	205 (38.3)	150 (31.7)	
**Meat n = 1008**			
Yes	493 (92.1)	443 (93.7)	0.35
No	42 (7.9)	30 (6.3)	
**Green Tea (100 ml)n = 1008**			
Yes	488 (91.2)	431 (91.1)	0.95
No	47 (8.8)	42 (8.9)	
**Arabic coffee (100 ml)n = 1008**			
Yes	447 (83.6)	414 (87.5)	0.07
No	88 (16.4)	59 (12.5)	
**Beverages (250 ml)n = 1008**			
Yes	97 (18.1)	127 (26.8)	<0.01
No	438 (81.9)	346 (73.2)	

Total number of patients with low BMD either at the lumbar spine or femoral neck using the DXA was 212 (58.5%) out of 362. Univariate analysis found age 60–70 years, education, personal history of fractures as an adult, husband occupation as an administrator and intake of dietary items; *low fat laban, green tea and Arabic coffee* during last one week as significantly associated with low BMD (Table 2). 

**Table 2:** Univariate regression analysis between socio demographic, menstrual status, medical and dietary items and low bone mineral density in women (> 40 years) in Riyadh, Saudi Arabia

**Characteristics**	**Women with Low BMD**	**Women with normal BMD**	**OR**
	**N = 212**	**N = 150**	**(95% CI)**
**Age (in years)n = 362**			
41 to 50	70 (33.2)	61 (41.2)	1.00
51 to 60	80 (37.9)	71 (48)	0.98 (0.61 - 1.56)
61 to 70	50 (23.7)	13 (8.8)	3.35 (1.66 - 6.75)
70 and above	11 (5.2)	3 (2)	3.19 (0.85 - 11.98)
**Marital Status n = 357**			
Single^2^	12 (6.2)	12 (7.3)	1.00
Married	135 (64.6)	101 (67.8)	0.89 (0.14 - 5.43)
Widow	60 (28.7)	37 (24.8)	1.08 (0.17 –6.77)
**Education n = 361**			
University and above	21 (9.9)	31 (20.8)	1.00
Intermediate& secondary	40 (18.9)	26 (17.4)	2.27 (1.08-4.77)
Primary	89 (42)	49 (32.9)	2.68 (1.39-5.15)
Illiterate	62 (29.2)	43 (28.9)	2.13 (1.08-4.18)
**Occupation n = 341**			
Doctor	2 (1.4)	1 (0.5)	1.00
Teacher/Administrator	19 (9.5)	16 (11.4)	2.37 (0.19 - 28.67)
Housewife	181 (90)	122 (87.1)	2.96 (0.26 - 33.08)
**Husbands Occupation n = 243**			
Doctors/teachers	11 (7.6)	17 (15.9)	1.00
Business man	47 (32.4)	33 (30.8)	2.20 (0.91 - 5.30)
Military	25 (17.2)	23 (210.5)	1.68 (0.65 - 4.32)
Administration	62 (42.8)	34 (31.8)	2.81 (1.18 - 6.70)
**Type of House n = 339**			
Villa	169 (85.4)	120 (85.1)	1.00
Apartment	19 (9.6)	13 (9.2)	1.03 (0.49 - 2.18)
Small house	10 (5.1)	8 (5.7)	0.88 (0.34 - 2.31)
**Body Mass Index n = 357**			
Normal	21 (10)	9 (6.1)	1.00
Overweight	61 (29)	36 (24.5)	0.72 (0.30 - 1.75)
Obese	128 (61)	102 (69.4)	0.53 (0.23 - 1.22)
**Menstrual status n = 329**			
Regular	70 (36)	55 (41)	1.00
Perimenopausal	69 (35.4)	48 (35.8)	1.13 (0.67 – 1.88)
Menopause	56 (28.6)	31 (23.2)	1.42 (0.80-2.49)
**Exposure to sunlight n = 341**			
All the time	45 (22.8)	39 (27)	1.00
Twice-thrice/week	49 (24.9)	34 (23.6)	1.24 (0.67 - 2.30)
Occasionally	83 (42)	58 (40.3)	1.24 (0.72 - 2.13)
Not at all	20 (10.2)	13 (9)	1.33 (0.58 - 3.02)
**Renal disease n = 362**			
No	200 (94.3)	148 (98.7)	1.00
Yes	12 (5.7)	2 (1.3)	4.44 (1.01 - 20.13)
**History of fractures as an adult on trivial fall n = 361**			
No	167 (84.3)	131 (92.3)	1.00
Yes	31 (15.7)	11 (7.7)	2.21 (1.07 - 4.56)
**Family History of fracture on trivial fall n = 287**			
No	155 (96.9)	124 (97.6)	1.00
Yes	5 (3.1)	3 (2.4)	1.33 (0.31 - 5.68)
**Eat Cheese (slice) 100 mg n = 362**			
Yes	178 (84)	125 (83.3)	1.00
No	34 (16)	25 (16.7)	0.95 (0.54 – 1.68)
**Drink Milk (200 ml) n = 362**			
Yes	167 (78.8)	120 (80)	1.00
No	45 (21.2)	30 (20)	1.07 (0.64 – 1.81)
**Drink Laban (200 ml) n = 362**			
Yes	163 (76.9)	135 (90)	1.00
No	49 (23.1)	15 (10)	2.70 (1.45 – 5.04)
**Drink Green Tea n = 362**			
**Yes**	185 (87.3)	140 (93.3)	1.00
**No**	27 (12.7)	10 (6.7)	2.04 (0.95 - 4.36)
**Drink Arabic coffee n = 362**			
**Yes**	177 (83.5)	137 (91.3)	1.00
**No**	35 (16.5)	13 (8.7)	2.08 (1.06 - 4.09)

Out of 535 women, 362 visited the hospital for DXA and 173 were missing. Women who did not get their DXA done reported transportation and male members’ unavailability as main factor for their absence. We compared the sociodemographic, health, dietary and physical activity data between those who got DXA done and not done. We found no significant difference (p > 0.05) among any of the variables except for husbands occupation (p < 0.01) between the two groups (results not shown).

In the final multivariate model, age 61 to 70 years (OR = 2.75 95%CI 1.32-1.48), no literacy (OR = 2.97, 95%CI 1.44-6.12), primary level of education (OR = 4.12 95%CI 2.05-8.29), history of personal fracture as an adult on trivial falls (OR = 2.20 95%CI 1.03-4.69) and not using laban in the diet (OR = 2.81 95%CI 1.47-5.37) were significantly associated with low BMD (Table 3). Physical activity was not associated with low BMD on univariate or multivariate analysis.

**Table 3:** Multivariate logistic regression showing adjusted odds ratio between age, educational level, dietary and medical factors with low bone mineral density in Saudi women (> 40 years) in Riyadh, Saudi Arabia

**Variables**	**Adjusted Odds Ratio**	**P value**
	**95% CI**	
**Level of Education**		
University and above	1.00	
Secondary& Intermediate	2.07 (1.06 - 4.55)	**0.04**
Primary	4.12 (2.05 - 8.29)	**0.02**
Illiterate	2.97 (1.44 - 6.12)	**0.009**
**Use of low fat laban in diet**		
Yes	1.00	
No	2.81 (1.47 - 5.37)	**0.008**
**Age (in years)**		
41 to 50	1.00	
51 to 60	0.88 (0.52-1.48)	**0.42**
61 to 70	2.75 (1.32-1.48)	**0.01**
70 and above	2.49 (0.63-9.89)	**0.18**
**History of fractures as an adult on trivial fall**		
Yes	1.00	
No	2.20 (1.03 - 4.69)	**0.03**

## Discussion

Previous research findings on high prevalence of low BMD in Saudi women are supported by this community-based study [4-7,9,14]. Majority of studies, however, have focused on postmenopausal women, while we included women 40 years and above because symptoms for low BMD tend to appear earlier in Saudi women [14]. Analogous to high burden, factors associated with low BMD like age, education and dietary products are in support of earlier studies [3,7-9,13-15] . Pathophysiology of aging in women indicates disconnection of trabecular network leading to reduction in bone mineral, structural deterioration and decrease in bone strength [16] which may explain the increase in prevalence of low BMD by 5% for each ten-year period between 40 and 50 years and 50 to 60 years. Old age effects on bones are further aggravated by estrogen deficiency due to menopause. The National Health and Nutrition Examination Survey (NHANES) for 2005–2008 reported an increase after 50 years of age in low BMD with each decade of age [3], hence supporting our results. Similarly, finding that being aged 60 to 70 years had the highest odds of low BMD is supported by NHANES results which found prevalence to increase until age 70 years, after which it remained static [3]. The recommended age for screening Saudi women remains controversial, some recommending 55 years [14] and others 65 years [15]; however, based on our findings we propose screening of all Saudi women for low BMD at age of 60 years. Social determinants of health, such as low education have been associated with low BMD [17]. Low education may prevent women from getting information on healthy life style habits including physical activity and access to health care thus augmenting the risk of low BMD.

Sedat et al. found incidence of vertebral fractures in Saudi Arabia between 20% and 24% [14]. One of the risk factors mentioned by National Osteoporosis Foundation in its guidelines for MENA region (Middle East and North Africa) is having personal history of fractures as an adult [8]. Fractures occurring during adulthood on exposure to some exercise, weight lifting or falls indicate weak bones and low bone mass density [18] and are a concern for future risk of osteoporosis [19]. Hence fractures and low bone density follow a viscous cycle; one leading to other and aggravating the condition further. Early screening of all such cases should be made mandatory as managing fractures in elderly is tedious and require support from family as well as health personal. In addition, the mortality, morbidity, disability and financial cost associated with management of fractures make screening for low BMD imperative [20,21].

Bone health is associated with diet rich in calcium and vitamin D, such as milk, cheese and laban (diluted yogurt) [22]. Milk and cheese, though consumed by majority after adjustment were not significantly associated with low BMD. However, “laban” also commonly known as “*lasi*” a dairy product manufactured by thermophilic fermented milk cultured with lactobacillus, bulgaricus and streptococcus thermophiles was significantly associated with low BMD. Several therapeutic benefits have been associated with fermented milk, [23,24] including decrease in bone deterioration, geriatric osteoporosis, skin ulcers, gastrointestinal symptoms and aging [24-27]. Quasi-experimental studies conducted on animal models have found fermented milk to reduce osteoporotic changes in mice [26,27]. Laban increases calcium assimilation [28]. Laban is a traditional drink of Saudi Arabia; however, its use seems to decrease with the introduction of new beverages. In view of present findings, we recommend regular use of laban as one of the preventive strategies against low BMD. As one cup (200 ml) of laban contains 238 mg of calcium and 80 IU of vitamin D, each women needs to take on an average 4–5 cups of laban per day in order to meet the recommended amount of calcium and vitamin D (daily recommended amount of Calcium 1000-1200 mg and vitamin D 600 IU) [29], therefore we conclude laban is an important and significant factor and even if taken alone in recommended amount can prevent low BMD. There may be other dietary sources contributing to blood vitamin D and calcium levels (for eg milk and cheese) and preventing low BMD but were not significant in our final model, for example, recently, use of green tea is proven to be protective against LBMD [30]. Future studies can recommend a dietary plan based on local food items to prevent women from developing low BMD.

This study is novel as it has used QUS as baseline screening test to identify “at risk” women before referring them for a much costlier and radiation exposed investigation, *DXA*. Recent studies found QUS to be a reliable screening instrument for low BMD/fractures and recommended its use in primary care settings [12,31,32]. Unnecessary exposure and cost related to DXA was reduced as 47% (473 women out of 1008) were normal on baseline screening and did not require undergoing DXA.

Vitamin D deficiency is supposed to identify at risk population for low BMD [33] but our results conclude that vitamin D may not be a reliable indicator for women in Saudi Arabia as majority were suffering from vitamin D deficiency, in fact low BMD women had higher values as compared to normal group.

### Limitations

Study had some limitations. 173 (32%) women did not undergo DXA testing. The main reason identified was limited mobility due to cultural factors. Although comparison of the characteristics of those tested and not tested with DXA revealed no significant difference except for husbands occupation, however, the groups may have differed on some characteristics that we did not ascertain. Several studies have taken cutoff point for QUS as t < −1; however, Sedat et al. recommended a different cut off [32] based on population differences, difference in results could be expected due to difference in cutoff. Women were labeled as menopausal and perimenopausal according to their responses and no laboratory investigation (FSH levels) was done to support their responses. Sun exposure was asked for current months only and type of clothing and seasonal variation was not taken into consideration. In addition, frequency of dietary items and physical activity may have been subjected to information bias. Thus, it is possible that residual uncontrolled confounding may have played a role in our findings.

## Conclusions

In conclusion, low BMD poses a high public health burden in Saudi Arabia which can be identified through screening and risk factors assessment at primary care level. Health education, regular screening and use of dietary products like laban (diluted yogurt) can help prevent low BMD in women.

## Competing interests

The authors declare that they have no competing interests.

## Authors’ contributions

Dr. JAQ was involved in conceptualization and write of proposal, conduction of the study and write up of the manuscript. Drs. AK, ST, SS, AAS and FH were involved in data collection, data cleaning, analysis and write up phase. Dr SO facilitated the conduction of study in King Khalid Hospital and was involved in the manuscript write up. Drs MF and RS monitored and facilitated the data collection process and reviewed the manuscript. All authors read and approved the final manuscript.

## Pre-publication history

The pre-publication history for this paper can be accessed here:

http://www.biomedcentral.com/1471-2474/15/5/prepub
